# Hemodynamic profiles of arterial hypertension with ambulatory blood pressure monitoring

**DOI:** 10.1038/s41440-023-01196-z

**Published:** 2023-03-08

**Authors:** Dagnovar Aristizábal-Ocampo, Diego Álvarez-Montoya, Camilo Madrid-Muñoz, Simon Fallon-Giraldo, Jaime Gallo-Villegas

**Affiliations:** 1 Centro Clínico y de Investigación SICOR, Medellín, Colombia; 2 Cellular & Molecular Biology Unit, Corporación para Investigaciones Biológicas, Medellín, Colombia; 3 Facultad de Medicina, Universidad de Antioquia, Medellín, Colombia

**Keywords:** Ambulatory blood pressure monitoring, Compliance, Hemodynamics, Hypertension, Vascular resistance

## Abstract

Blood pressure (BP) measurements obtained during a twenty-four-hour ambulatory blood pressure monitoring (24 h ABPM) have not been reliably applied to extract arterial hemodynamics. We aimed to describe the hemodynamic profiles of different hypertension (HT) subtypes derived from a new method for total arterial compliance (C_t_) estimation in a large group of individuals undergoing 24 h ABPM. A cross-sectional study was conducted, which included patients with suspected HT. Cardiac output, C_t_, and total peripheral resistance (TPR) were derived through a two-element Windkessel model without having a pressure waveform. Arterial hemodynamics were analyzed according to HT subtypes in 7434 individuals (5523 untreated HT and 1950 normotensive controls [N]). The individuals mean age was 46.2 ± 13.0 years; 54.8% were male, and 22.1% were obese. In isolated diastolic hypertension (IDH), the cardiac index (CI) was greater than that in normotensive (N) controls (CI: IDH vs. N mean difference 0.10 L/m/m^2^; CI 95% 0.08 to 0.12; *p* value <0.001), with no significant clinical difference in C_t_. Isolated systolic hypertension (ISH) and divergent systolic-diastolic hypertension (D-SDH) had lower C_t_ values than nondivergent HT subtype (C_t_: divergent vs. nondivergent mean difference −0.20 mL/mmHg; CI 95% −0.21 to −0.19 mL/mmHg; *p* value <0.001). Additionally, D-SDH displayed the highest TPR (TPR: D-SDH vs. N mean difference 169.8 dyn*s/cm^−5^; CI 95% 149.3 to 190.3 dyn*s/cm^−5^; *p* value <0.001). A new method is provided for the simultaneous assessment of arterial hemodynamics with 24 h ABPM as a single diagnostic tool, which allows a comprehensive assessment of arterial function for hypertension subtypes.

**Figure:**
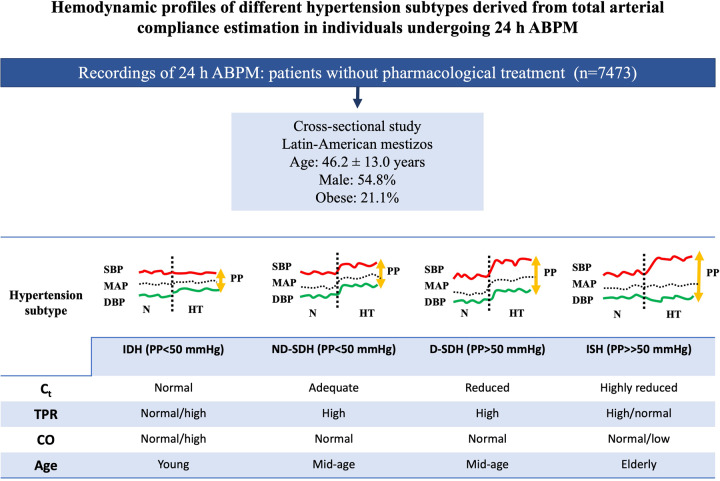
Main hemodynamic findings in arterial HT subtypes with regard to C_t_ and TPR. The 24 h ABPM profile reflects the state of C_t_ and TPR. Younger individuals with IDH present with a normal C_t_ and frequently increased CO. Patients with ND-SDH maintain an adequate C_t_ with a higher TPR, while subjects with D-SDH present with a reduced C_t_, high PP and high TPR. Finally, the ISH subtype occurs in older individuals with significantly reduced C_t_, high PP and a variable TPR proportional to the degree of arterial stiffness and MAP values. There was an observed increase in PP with age in relation to the changes in C_t_ (see also text). SBP: systolic blood pressure; DBP: diastolic blood pressure; MAP: mean arterial pressure; PP: pulse pressure; N: normotension; HT: hypertension; IDH: isolated diastolic hypertension; ND-SDH: nondivergent systole-diastolic hypertension; D-SDH: divergent systolic-diastolic hypertension; ISH: isolated systolic hypertension; C_t_: total arterial compliance; TPR: total peripheral resistance; CO: cardiac output; 24 h ABPM: 24 h ambulatory blood pressure monitoring.

Main hemodynamic findings in arterial HT subtypes with regard to C_t_ and TPR. The 24 h ABPM profile reflects the state of C_t_ and TPR. Younger individuals with IDH present with a normal C_t_ and frequently increased CO. Patients with ND-SDH maintain an adequate C_t_ with a higher TPR, while subjects with D-SDH present with a reduced C_t_, high PP and high TPR. Finally, the ISH subtype occurs in older individuals with significantly reduced C_t_, high PP and a variable TPR proportional to the degree of arterial stiffness and MAP values. There was an observed increase in PP with age in relation to the changes in C_t_ (see also text). SBP: systolic blood pressure; DBP: diastolic blood pressure; MAP: mean arterial pressure; PP: pulse pressure; N: normotension; HT: hypertension; IDH: isolated diastolic hypertension; ND-SDH: nondivergent systole-diastolic hypertension; D-SDH: divergent systolic-diastolic hypertension; ISH: isolated systolic hypertension; C_t_: total arterial compliance; TPR: total peripheral resistance; CO: cardiac output; 24 h ABPM: 24 h ambulatory blood pressure monitoring.

## Abstract (web summary)

Main hemodynamic findings in arterial HT subtypes with regard to C_t_ and TPR. The 24 h ABPM profile reflects the state of C_t_ and TPR. Younger individuals with IDH present with a normal C_t_ and frequently increased CO. Patients with ND-SDH maintain an adequate C_t_ with a higher TPR, while subjects with D-SDH present with a reduced C_t_, high PP and high TPR. Finally, the ISH subtype occurs in older individuals with significantly reduced C_t_, high PP and a variable TPR proportional to the degree of arterial stiffness and MAP values. There was an observed increase in PP with age in relation to the changes in C_t_ (see also text). SBP: systolic blood pressure; DBP: diastolic blood pressure; MAP: mean arterial pressure; PP: pulse pressure; N: normotension; HT: hypertension; IDH: isolated diastolic hypertension; ND-SDH: nondivergent systole-diastolic hypertension; D-SDH: divergent systolic-diastolic hypertension; ISH: isolated systolic hypertension; C_t_: total arterial compliance; TPR: total peripheral resistance; CO: cardiac output; 24 h ABPM: 24 h ambulatory blood pressure monitoring.

## Introduction

Twenty**-**four-hour blood pressure monitoring (24 h ABPM) has an established role in evaluating and managing arterial hypertension (HT). Current HT guidelines accept 24 h ABPM as the method of choice for a precise diagnosis and evaluation of HT [1, 2] and confirm various phenotypes based on ambulatory blood pressure (BP) behavior. To date, however, oscillometric BP measurements obtained during a 24 h ABPM study have not been consistently applied to extract hemodynamic parameters such as total arterial compliance (C_t_), cardiac output (CO), or total peripheral resistance (TPR).

Some of the hemodynamic abnormalities observed in HT are attributed to an increased TPR or a low C_t_ [3, 4], and their identification helps to understand the pathophysiology of essential HT and may also facilitate patient management [5]. Nevertheless, the obtention of C_t_ and other hemodynamic parameters could need dedicated instrumentation or devices which require trained observers [3, 6–8]. Previous clinical studies have shown the importance of C_t_ and its effects on aortic pulsatility and pulse pressure (PP) to understand the pathogenesis of arterial hypertension [9, 10]. However, the inclusion of C_t_ in clinical studies of hypertension hemodynamics has been limited. Recently, Majajan et al. [6] evaluated a large group of hypertensive outpatients using impedance cardiography to obtain the cardiac index (CI) and systemic vascular resistance index but not C_t_.

Great heterogeneity was observed in the hemodynamic phenotype between patients with similar degrees of BP elevation, with no clear explanation for those findings [6] Also, studies of the isolated systolic hypertension (ISH) subtype, have shown contrasting differences in age and anthropometric factors [11*–*13] as well as in aortic properties which are linked to arterial compliance changes and affect the BP profile [6, 10]. Those observations emphasize the need of clinical methods to assess simultaneously steady and pulsatile hemodynamics.

The possibility of deriving hemodynamic features with the same BP values obtained during a 24 h ABPM study would refine this method by adding complementary pathophysiological information about the function of the arterial system in hypertensive patients [14]. We aimed to describe the hemodynamic profiles of different HT subtypes derived from this new C_t_ estimation method obtained from the average ambulatory BP and heart rate (HR) values in a large group of individuals undergoing 24 h ABPM. We hypothesize that there are different hemodynamic profiles according to the HT subtypes.

## Methods

### Study design

A cross-sectional study was conducted, which included patients with suspected BP abnormalities who were referred to the Centro Clinico y de Investigacion SICOR, Medellín (Colombia), to undergo a 24 h ABPM between November 2017 and November 2021.

### Study population

The population was conformed by Latin-American individuals with the vast majority being mestizos. Patients were referred by independent physicians mainly due to suspicion of HT. Patients with missing data, unsatisfactory 24 h ABPM recordings and under the age of 18 were excluded. Unsatisfactory recordings were considered when less than 40 measurements or more than 100 measurements were obtained. Additionally, whenever a difference between two measurements was greater than 2 h, the study lasted less than 18 h or more than 24 h, and if BP measurements were less than 30 during the day and less than 10 or more than 25 during the night.

### 24 h ambulatory blood pressure monitoring assessment

Each patient was put on a validated Custo-Screen® (Custo, Munich, Germany) 24 h ABPM device [15]. Before the installation, demographic and clinical information, habits, and anthropometric measurements were recorded following a predefined protocol [16]. Cuffs were selected based on the diameter of the nondominant arm. Measurements of BP were obtained every 15 min throughout the day (6:00–22:00) and every 30 min throughout the night (22:00–6:00) [1]. Normotension was defined as a 24 h mean diastolic blood pressure (DBP) < 80 mmHg and a 24 h mean systolic blood pressure (SBP) < 130 mmHg [1]. Isolated diastolic hypertension (IDH) was defined as DBP ≥ 80 mmHg with SBP < 130 mmHg. When SBP was ≥130 mmHg, and DBP ≥ 80 mmHg, systolic-diastolic hypertension (SDH) subtype was defined. Based on previous clinical studies, the lower risk quartile and percentile for PP was 50 mmHg [17, 18]. Thus, this value was selected as a cut-off point for a normal pulse pressure. Then, SDH subtype was subdivided into two categories: divergent systolic-diastolic hypertension (D-SDH) if pulse pressure (PP) ≥ 50 mmHg and nondivergent systolic-diastolic hypertension (ND-SDH) in case PP < 50 mmHg. Finally, ISH was defined as SBP > 130 mmHg with DBP < 80 mmHg. Mean arterial pressure (MAP) (mmHg) was calculated as DBP + PP/3. All data were stored in the institutional database.

### Estimation of total arterial compliance and cardiac output with oscillometric blood pressures

In a previous study, we developed and validated a mean systolic pressure (MSP) method for estimating C_t_ and CO without a BP waveform analysis through a two-element Windkessel (2-WK) model) [14]. MSP is a measurement of the volume transfer to the aorta in each heartbeat in terms of pressure, which is estimated as (MAP+SBP)/2. This formula is obtained with a trapezoid built under the systolic area of an arterial pressure curve and employing SBP, MAP and systolic time (T_s_) [14].

C_t_ was defined as the ratio between aortic systolic volume and the theorical pulse pressure (PPth) (i.e., the aortic pressure value which could be reached if the systolic runoff had been prevented during aortic ejection) (Supplementary Methods 1). Experimentally, the PPth is the “true” aortic pressure needed to obtain the C_t_ (Supplementary Methods 2) [19]. In our calculations, this value could be obtained with the following equation: (Supplementary Methods 1).

$$C_t = \frac{{SV}}{{PP + MSP \times \frac{{T_s}}{\tau }}}$$

Where, C_t_, total arterial compliance; SV, stroke volume; PP, pulse pressure; MSP, mean systolic pressure (the mean pressure during the ejection period); T_s_, systolic time; and τ, diastolic pressure decay time constant.

The stroke volume (SV) obtained with echocardiography in two hundred and thirty consecutive patients was used to calculate C_t_ and a regression model for C_t_ was developed and applied to estimate CO. For C_t_ assessment, a parsimonious model based on the 2-WK was incorporated by changing the exponential approximation for the time constant of diastolic pressure decay (τ) [20] with a new estimate based on the ratio of steady and the pulsatile components of BP (Supplementary Methods 3) [19].

Then, Ct was estimated as:

$$\frac{{C_t}}{{BSA}} = \frac{{38}}{{PPth}} + \frac{4}{5} \times \frac{{T_d}}{T} - \frac{3}{7}$$

With, Ct, total arterial compliance; BSA, body surface area; Td, diastolic time; T, cardiac period; PPth, theoretical pulse pressure (Supplementary Methods 2).

### Habits

The levels of physical activity were defined as follows: i) level I as inactive or exercise of less than one hour a week; ii) level II as participating in moderate-intensity exercise between one to less than three hours per week; and iii) level III as exercise equal to or greater than three hours per week. A smoker was defined as a current smoker, and liquor intake was considered frequent if consumed at least once a week.

### Statistical analysis

Initially, an exploratory analysis of the data was conducted to detect errors in the coding of the variables, including possible inconsistencies in the data, lost data, and outliers. This analysis also allowed us to become familiar with the basic distribution characteristics of the variables. Graphical methods (histogram and normal probability plot) were used to evaluate whether the variables came from a normally distributed population. The variables that correlated with the body surface area (BSA) were indexed [21]. For the positive correlation, the variable was divided by the BSA and multiplied if negatively correlated [22]. The variables indexed to BSA were SV, CO, C_t_, and TPR. The means, standard deviations, medians, and percentiles were used to describe the quantitative variables. Proportions were utilized to describe the categorical variables. One-factor ANOVA with Bonferroni correction was used to compare the quantitative variables between the subgroups, while the chi-square test of independence was employed to compare categorical variables. The results are presented as the mean differences with their respective confidence intervals of 95%; an alpha error of 0.05 was considered. Analyses were conducted using STATA^®^, v.14.0 (StataCorp LLC, Texas, United States) and MATLAB^®®^, v.9.6 (The MathWorks, Inc., Massachusetts, United States).

### Ethical aspects

The Institutional Ethics Committee approved this study. Prior to installing the equipment for the diagnostic evaluation, informed written consent was obtained to use the examination results for research purposes. The health research rules of Resolution 008430 of 1993 from the Ministry of Social Protection of Colombia and the principles of the Declaration of Helsinki in its latest revision were considered [23].

## Results

**Fig. 1:**
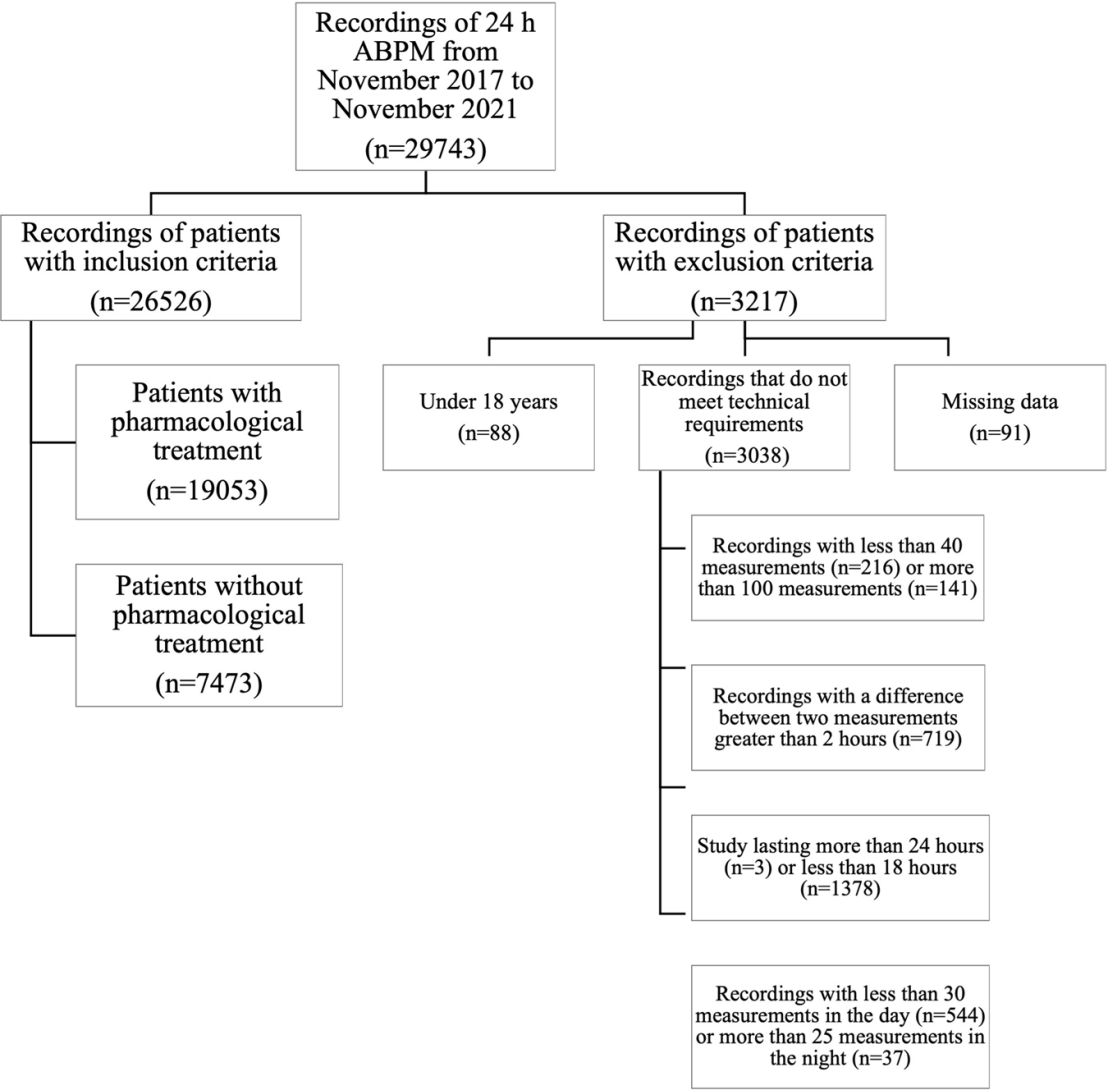
Flowchart showing how 24 h ambulatory blood pressure monitoring recordings were included. 24 h ABPM: 24 h ambulatory blood pressure monitoring

**Fig. 2:**
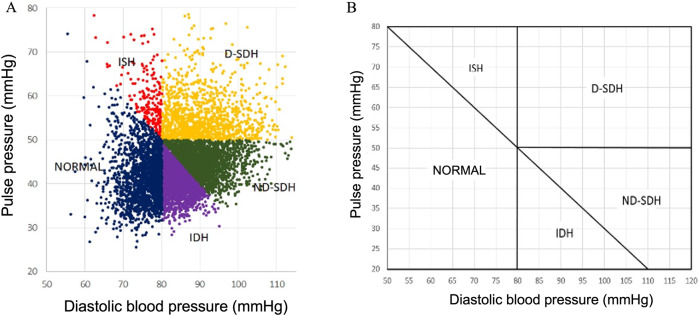
Relationship between diastolic blood pressure (DBP) and pulse pressure (PP) with hypertension subtypes in 24 h ambulatory blood pressure monitoring in 7473 subjects without pharmacologic treatment (**A**). Normotensive and hypertension subtypes zones were obtained according to the definition for elevated systolic and diastolic blood pressure as it is presented in method´s section. The diagonal line defines the border between normal and high systolic blood pressure (SBP), with all subjects above the diagonal line with systolic hypertension. The vertical line defines the limit for normal DBP (80 mmHg). Systolic hypertension (above the diagonal line) and diastolic hypertension (right side of the vertical line) define zones with either one elevated blood pressure (isolated systolic hypertension [ISH] and isolated diastolic hypertension [IDH]) or with both blood pressures elevated (non-divergent systolic and diastolic hypertension [ND-SDH] and divergent systolic and diastolic hypertension [D-SDH]) (**B**). NORMAL: DBP < 80 mmHg and SBP < 130 mmHg; ISH: Isolated systolic hypertension, SBP > 130 mmHg, DBP < 80 mmHg and, PP ≥ 50 mmHg; IDH: Isolated diastolic hypertension, SBP < 130 mmHg, DBP ≥ 80 mmHg and, PP < 50 mmHg; ND-SDH: Non-divergent systolic and diastolic hypertension, SBP ≥ 130 mmHg, DBP ≥ 80 mmHg and, PP < 50 mmHg; D-SDH: Divergent systolic and diastolic hypertension, SBP ≥ 130 mmHg, DBP ≥ 80 mmHg and, PP ≥ 50 mmHg

**Fig. 3:**
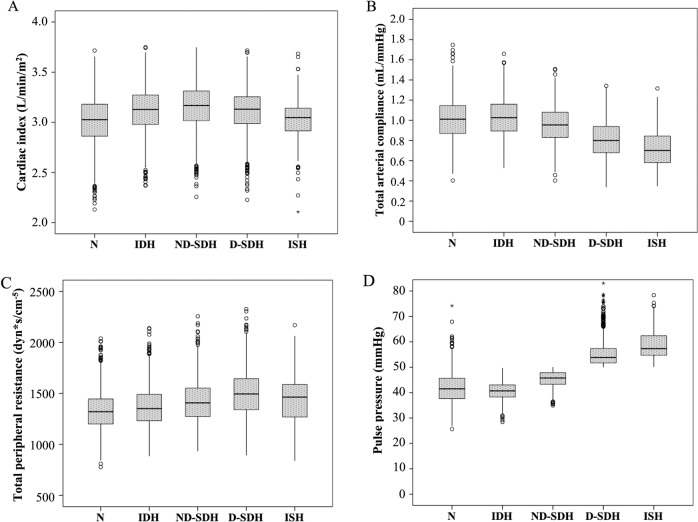
Box-and-whisker plot showing the distribution of cardiac index, total peripheral resistance, total arterial compliance, and pulse pressure according to arterial hypertension categories in the 24 h ambulatory blood pressure monitoring in the patients without pharmacological treatment. The cardiac index is stable (between 3.01 and 3.16 L/min/m^2^ from N to ISH), but TPR increased from N state to D-SDH (N, IDH, ND-SDH, and D-SDH were 1329.3, 1365.7, 1422.5 and 1499.1 dyn*s/cm^−5^, respectively; TPR: D-SDH vs. N mean difference 169.8 dyn*s/cm^−5^; CI 95% 149.3 to 190.3 dyn*s/cm^-5^; *p* value<0.001); only in ISH was a reduction observed (1441.6 dyn*s/cm^-5^; TPR: ISH vs. D-SDH mean difference −57.5; CI 95% −100.0 to −14.9 dyn*s/cm^−5^; *p* value = 0.001). On the other hand, C_t_ is reduced progressively from IDH to ISH (1.01 to 0.72 mL/mmHg; C_t_: ISH vs. IDH mean difference −0.31 mL/mmHg; CI 95% −0.35 to −0.27 mL/mmHg; *p* value < 0.001); PP is the lowest in IDH (40.6 ± 3.5 mmHg), while it increases in all the remaining hypertension subtypes (PP: ISH vs. IDH mean difference 18.5 mmHg; CI 95% 17.6 to 19.5 mmHg; *p* value < 0.001). N: Normotensive; IDH: Isolated diastolic hypertension; ND-SDH: Nondivergent systolic-diastolic hypertension; D-SDH: Divergent systolic-diastolic hypertension; ISH: Isolated systolic hypertension; TPR: total peripheral resistance; C_t_: total arterial compliance; PP: Pulse pressure. The *p* value in one-factor ANOVA was <0.001 for the cardiac index, total peripheral resistance, total arterial compliance, and pulse pressure

Of 29,743 consecutive patients who underwent a 24 h ABPM, 26,526 (89.2%) met the inclusion criteria. Due to missing data (*n* = 91), studies that did not meet technical requirements (*n* = 3038) or patients under 18 years (*n* = 88), 10.8% of all 24 h ABPMs were excluded. Among all patients with inclusion criteria (*n* = 26,526), 7473 subjects (28.2%) who did not receive any type of pharmacological treatment were analyzed (Fig. 1). The demographic, anthropometric and clinical data of all analyzed patients are shown in Table 1. The individuals analyzed mean age was 46.2 ± 13.0 years; 54.8% were male, and 22.1% were obese. Nondivergent subgroups, which included IDH and ND-SDH, were younger than divergent subgroups (D-SDH and ISH) (*p* value<0.001). Figure 2 presents the HT subtypes distribution according to the systolic and diastolic BP and, PP cut-off values. The most common subtype was IDH (28.2%). In the IDH subtype, the CI was greater than in normotensive (N) controls (CI: IDH vs. N mean difference 0.10 L/m/m^2^; CI 95% 0.08 to 0.12; *p* value < 0.001), with no significant clinical difference in C_t_ (C_t_: IDH vs. N mean difference 0.02 mL/mmHg; CI 95% 0.01 to 0.03; *p* value 0.009) (Table 1, Fig. 3A, B). Divergent HT subtypes (ISH and D-SDH) had a statistically significant lower C_t_ than nondivergent HT subtypes (IDH and ND-SDH) (C_t_: divergent vs. nondivergent mean difference −0.20 mL/mmHg; CI 95% −0.21 to −0.19 mL/mmHg; *p* value<0.001) (Table 1 and Fig. 3B). In addition, the D-SDH subtype showed the highest TPR (TPR: D-SDH vs. *N* mean difference 169.8 dyn*s/cm^−5^; CI 95% 149.3 to 190.3 dyn*s/cm^−5^; *p* value<0.001) (Table 1 and Fig. 3C). Finally, the ISH subtype despite being older (58.6 ± 19.7 years) with the largest reduction in C_t_ (0.72 ± 0.19 mL/mmHg), this subgroup showed less TPR than D-SDH. (TPR: ISH vs. D-SDH mean difference −57.5 dyn*s/cm^−5^; CI 95% −100.0 to −14.9 dyn*s/cm^−5^; *p* value=0.001) (Table 1, Fig. 3B, C).

**Table 1:** Demographic, anthropometric, clinical characteristics and haemodynamic variables were extracted from 24 h ambulatory blood pressure monitoring patients without pharmacological treatment

Patients without pharmacological treatment *n* = 7473						
Variables	Normotensive	IDH	ND-SDH	D-SDH	ISH	*P* value
	*n* = 1950 (26.1%)	*n* = 2109 (28.2%)	*n* = 2005 (26.8%)	*n* = 1207 (16.2%)	*n* = 202 (2.7%)	
	Mean SD [p5, p95]	Mean SD [p5, p95]	Mean SD [p5, p95]	Mean SD [p5, p95]	Mean SD [p5, p95]	
Age (years)	45.0 ± 14.2 [23.1, 68.7]	44.7 ± 10.9 [27.3, 63.3]	45.4 ± 10.8 [28.3, 63.1]	50.3 ± 14.6 [24.7, 73.3]	58.6 ± 19.7 [22.1, 85.3]	<0.001
Body mass index (kg/m^2^)	26.1 ± 3.8 [20.2, 32.9]	26.8 ± 3.7 [21.2, 33.5]	27.9 ± 3.9 [22.0, 34.9]	28.4 ± 4.4 [22.2, 36.4]	27.1 ± 4.1 [21.3, 35.7]	<0.001
Body surface area (m^2^)	1.81 ± 0.20 [1.50, 2.18]	1.86 ± 0.20 [1.53, 2.20]	1.92 ± 0.21 [1.57, 2.27]	1.91 ± 0.23 [1.55, 2.28]	1.80 ± 0.23 [1.46, 2.19]	<0.001
Heart rate (beats per min)	72.8 ± 9.1 [57.8, 87.8]	76.6 ± 8.6 [63.0, 90.7]	77.2 ± 9.3 [62.2, 92.3]	73.7 ± 9.2 [58.9, 88.6]	70.8 ± 9.5 [55.7, 89.6]	<0.001
Systolic blood pressure (mmHg)	117.1 ± 6.8 [104.7, 127.7]	124.6 ± 3.7 [117.5, 129.5]	136.6 ± 5.5 [130.4, 148.0]	146.4 ± 9.5 [133.8, 163.2]	135.8 ± 5.0 [130.3, 146.7]	<0.001
Diastolic blood pressure (mmHg)	75.2 ± 3.9 [67.9, 79.6]	84.0 ± 2.7 [80.3, 88.0]	91.2 ± 5.4 [83.2, 101.6]	90.9 ± 7.6 [81.2, 105.0]	76.6 ± 3.3 [69.5, 79.8]	<0.001
Mean arterial pressure (mmHg)	89.2 ± 4.2 [81.4, 94.6]	97.5 ± 2.7 [93.3, 102.0]	106.3 ± 5.3 [99.5, 116.8]	109.4 ± 7.9 [99.0, 123.5]	96.3 ± 2.7 [91.5, 100.2]	<0.001
Pulse pressure (mmHg)	41.8 ± 5.8 [33.3, 51.6]	40.6 ± 3.5 [34.6, 46.3]	45.4 ± 3.0 [39.9, 49.5]	55.5 ± 5.4 [50.3, 66.2]	59.1 ± 6.2 [51.6, 72.8]	<0.001
Stroke volume/BSA (mL/m^2^)	41.7 ± 2.2 [38.1, 45.2]	40.9 ± 1.9 [37.6, 43.9]	41.2 ± 2.3 [37.3, 44.6]	42.5 ± 2.8 [37.7, 46.7]	43.1 ± 3.2 [38.1, 47.9]	<0.001
Cardiac index (L/min/m^2^)	3.01 ± 0.24 [2.57, 3.39]	3.12 ± 0.22 [2.74, 3.45]	3.16 ± 0.22 [2.75, 3.49]	3.11 ± 0.22 [2.72, 3.45]	3.03 ± 0.23 [2.64, 3.41]	<0.001
Total arterial compliance (mL/mmHg)	1.01 ± 0.20 [0.68, 1.34]	1.03 ± 0.18 [0.74, 1.33]	0.95 ± 0.17 [0.67, 1.23]	0.80 ± 0.18 [0.52, 1.10]	0.72 ± 0.19 [0.42, 1.10]	<0.001
Total peripheral resistance (dyn*s/cm−5)	1329 ± 183 [1045,1644]	1365 ± 186 [1087,1686]	1422 ± 205 [1123,1793]	1499 ± 228 [1140,1906]	1441 ± 227 [1056,1799]	<0.001

		*n*	%	*n*	%	*n*	%	*n*	%	*n*	%	
Men		856	44	1162	55	1302	65	697	58	79	39	<0.001
Level of physical activity	I	1176	60	1329	63	1317	66	759	63	123	61	0.015
	II	448	23	449	21	410	20	248	21	45	22	
	III	326	17	331	16	278	14	200	17	34	17	
Frequent alcohol intake		211	11	308	15	347	17	216	18	24	12	<0.001
Current smoking		92	5	121	6	140	7	99	8	16	8	0.001

*IDH* Isolated diastolic hypertension, *ND-SDH* Nondivergent systolic-diastolic hypertension. *D-SDH* Divergent systolic-diastolic hypertension, *ISH* Isolated systolic hypertension, *BSA* Body surface area

## Discussion

Our study presents for the first time the clinical implementation of a simple and accurate method to assess arterial hemodynamics with 24 h ABPM data. The main findings of this study are that both C_t_ and TPR are the key hemodynamic parameters responsible for the 24 h ABPM profile. When BP increases without changes in HR, TPR is the main reason for BP elevation, and BP rises with a nondivergent HT subtype (i.e., PP < 50 mmHg). On the other hand, if both pressures are elevated with a divergent HT subtype (i.e., PP ≥ 50 mmHg), a reduction in C_t_ also occurs in addition to TPR elevation. Most importantly, an IDH subtype is present when C_t_ is normal. Finally, the ISH subtype characteristically has the largest reduction in C_t_ with the greatest divergence in SBP and DBP (Fig. 3B, D).

Altogether, the present work provides a generalizable method to track arterial hemodynamics in parallel with BP and HR values obtained with a 24 h ABPM. We are not aware of any study reporting a mathematical model to build a good estimate of C_t_, CO and TPR with only 24 h ABPM data. To disclose such a hemodynamic information, other methods such as impedance cardiography, echocardiography or magnetic resonance images are normally required. With the current approach, hemodynamic information is simultaneously derived from oscillometric BP values.

Mathematical models of arterial hemodynamics have been employed for more than a century [24]. A simple model helps to understand the most influential features of circulatory physiology [25*–*27]. In addition, these simple models employ only a few parameters that can be reliably estimated from the limited measurements available in clinical practice [26, 28]. One of those key parameters is arterial compliance, which allows blood flow and pressure to occur with less oscillation between systolic and diastolic pressures. However, as direct measurement of arterial compliance in humans in vivo is very difficult to obtain, the analysis of the importance of C_t_ to understand BP behavior over the whole range of HT subtypes has been limited. With the provision of a clinically applicable estimate of C_t,_ this analysis was possible in the present study. Our findings corroborate and add to previous observations indicating that a low C_t_ causes a significant divergence in BP values [3, 29–32]. On the other hand, a normal C_t_ maintains SBP and DBP closer with a low PP (Fig. 3). The relevance of those BP effects is significant. Several physiological and longitudinal clinical studies have demonstrated that a low C_t_ indicates an increased arterial stiffness, which provokes a PP increment and is accompanied by a greater cardiovascular risk [33*–*36]. Also, our findings are in agreement with recent observations indicating that load-dependent stiffness mediated by high BP values is more important than structural stiffness to determine cardiovascular risk [37]. In contrast, IDH, characterized by high compliance and low arterial stiffness, has a lower cardiovascular risk [38*–*40], particularly in subjects over 50 years old [41]. In this sense, our results provide complementary insights into the physiological explanation for the lower risk of IDH.

The classic understanding of hemodynamics in HT has emphasized the steady flow component, where the heart behaves as a pump providing continuous blood flow through the aorta and the arterial system is considered a resistor that opposes the flow of blood. Thus, the MAP, determined by the mean flow and the mean resistance of the arterial system, is the steady-state component of arterial pressure [5]. However, the episodic nature of cardiac contraction and the properties of the arterial circulation create BP oscillation with PP, reflecting the effects of the pulsatile conditions. To integrate the effects of these two components (steady and pulsatile) on BP behavior, the 2-WK model simplifies the circulation in terms of resistance and capacitance components [24]. The resistance component corresponds to TPR, while the capacitance component represents C_t_ [33]. Early studies have shown that a 2-WK model outperforms other lumped models to evaluate C_t_ [42], and therefore, for this purpose, the 2-WK model is preferred [26, 43–45]. Previously, Stergiopolus experimentally demonstrated that with both C_t_ and TPR, the behavior of BP could be determined [26]. However, a clinical demonstration of BP response along all subtypes of HT with 24 h ABPM has not yet been presented. Our method emphasizes the importance of systolic pulsatile BP behavior to identify the state of C_t_ according to the HT subtype in a 24 h ABPM. These findings demonstrate how a reduction in C_t_ is typically detected by a higher PP with a divergent BP pattern in 24 h ABPM (Table 1). A previous study by Scuteri et al. [46] found that increased aortic stiffness (a sign of reduced arterial compliance) is implicated in the age-associated rise in SBP and PP; such a finding was noticed in our patients with D-SDH and ISH subtypes (Table 1). Similar findings were also reported by Krzesinski et al. employing impedance cardiography [31]. Instead, younger individuals showed a preserved C_t_ and characteristically presented with IDH or ND-SDH subtypes (Table 1).

According to our findings not all elderly subjects have the same HT subtype (e.g., isolated systolic hypertension). Age effects on arterial pressure profile (and HT subtype) are highly influenced by C_t_ values and those effects are not uniform during aging. Old subjects with a fair arterial compliance could have a lower PP than younger counterparts who have a compromised arterial compliance (Table 2 and Supplementary Fig. 1). Those clinical finding only appear when hemodynamics of HT is described based on BP subtypes and with the inclusion of C_t_. Thus far, HT continues to be seen as a condition due to either high TPR or high CO. The relevance of C_t_ in those analysis has been marginal. By simultaneously assessing the hemodynamics of HT subtypes with 24 h ABPM, we were able to notice the differences in arterial behavior of these HT subtypes, which could have pathophysiological meaning for HT. For instance, elderly hypertensives may be wrongly assumed as having structural arterial stiffness when C_t_ is unknown. On the other hand, middle-aged individuals not always have an increased TPR, in particular those with IDH. Those observations are novel pieces of clinical information which were not feasible to recognize in previous studies [13].

**Table 2:** Step by step calculations with a case study example

Parameter	Calculation	Result	Comments
Gender	Male = 1, Female = 0	1	
Age		71 years old	
Weight		72 Kg	
Height		1.75 m	
BSA	$BSA = \sqrt {weight \times height \times 100 \div 3600}$	1.87 Kg/mt^2^	Reference [21]
HR		76 bpm	Average value in 24 h ABPM
SBP		132 mmHg	Average value in 24 h ABPM
DBP		86 mmHg	Average value in 24 h ABPM
PP	*PP*=*SBP* - *DBP*	46 mmHg	Standard calculation
MAP	*MAP*=*DBP*+0.33×*PP*	101 mmHg	Standard calculation
MSP	$MSP = \frac{{SBP + MAP}}{2}$	117 mmHg	See methods section
T	$T = \frac{{60}}{{HR}}$	0.789 s	Standard calculation
T_s_	$T_s = 0.2061 + \frac{{{{{{{{{\mathrm{Age}}}}}}}}}}{{2244}} - \frac{{Gender}}{{72.39}} + \frac{T}{{8.17}}$	0.324 s	Supplementary Methods 3, equation 12
T_d_	*T*_*d*_ = *T* – *T*_*s*_	0.469 s	Standard calculation
Tau (*τ*)	$\tau = \frac{{MAP \times T - MSP \times T_s}}{{PP}}$	0.908 s	Supplementary Methods 2, equation 9
PPth	$PPth = PP + \left( {MSP \times \frac{{T_s}}{\tau }} \right)$	88 mmHg	Supplementary Methods 2, equation 4
C_t_/BSA*	$\frac{{C_t}}{{BSA}} = \frac{{38}}{{PPth}} + \frac{4}{5} \times \frac{{T_d}}{T} - \frac{3}{7}$	0.479 (ml/mmHg)/m^2^	Methods section
SV	$SV = C_t\left( {PP + MSP \times \frac{{T_s}}{{{{{{{{\mathrm{\tau }}}}}}}}}} \right)$	79 ml	Supplementary Methods 1, equation 4
CO	*CO*=*SV*×*HR*	6.0 L/min	Standard calculation
TPR	$TPR = \frac{{MAP}}{{CO}} \times 80$	1347 dynes. s. cm^−5^	Standard calculation

A 71 y/o asymptomatic male attends an annual health check-up. Baseline blood pressure was normal; however, he had a hypertensive response during the treadmill stress test. A 24 h ambulatory blood pressure monitoring was indicated to clarify a possible masked hypertension. BSA: Body surface area; HR: Heart rate; 24 h ABPM: 24 h ambulatory blood pressure monitoring; Systolic blood pressure; DBP: Diastolic blood pressure; PP: Pulse pressure; MAP: Mean arterial pressure; MSP: Mean systolic pressure; T: Cardiac period; T_s_: Systolic time; T_d_: Diastolic time; Tau (τ): time constant of diastolic pressure decay; PPth: Theoretical pulse pressure; C_t_/BSA: Total arterial compliance index; SV: Stroke volume; CO: Cardiac output; TPR: Total peripheral resistance. *To convert C_t_/BSA to usual units, multiply for BSA, then C_t_ = 0.89 mL/mmHg

In a preceding study, Galarza et al. [3] compared the hemodynamic changes in different age groups and pressure patterns by employing impedance cardiography to obtain systemic hemodynamics. They observed a sustained increase in TPR with age from the third to the seventh decade. Interestingly, their older subjects presented with ISH with a combination of reduced C_t_ and a high TPR. As shown in Fig. 3, this hemodynamic pattern was consistently observed in our hypertensive subgroups from IDH through ISH. In addition, with our method based only on 24 h ABPM, we arrived at the same conclusion as Galarza et al. [3] to explain how a reduction in C_t_ with age may counteract the effect of a TPR increment on DBP (Table 1).

Given the relevance of large arteries in BP behavior, our C_t_ estimation method adds mechanistic insights into the hemodynamic basis of HT and their relationship with the 24 h ABPM profile. A high TPR caused by arteriolar vasoconstriction has traditionally been viewed as the key determinant of MAP and DBP increase [47, 48]. On the other hand, aortic stiffening is responsible for the SBP elevation, mainly in elderly subjects [48, 49]. More recent evidence has suggested that PP analysis provides additional pathophysiological meaning to understanding the conditions of the arterial circulation [34, 39, 50]. It is noticeable that regardless of age, an increase in PP signals a reduction in C_t_ (Table 1 and Fig. 3), a finding also described in previous clinical studies [50, 51], which indicates the significant role played by aortic function in the pathophysiology of systolic HT. Interestingly, a reduction in C_t_ with an increased PP could occur without structural degeneration of the aorta, due to load-dependent stiffness. That is why systolic HT is seen in middle-aged individuals [52] and the systolic pressure raises in the pediatric obese population [53]. Nonetheless, in old individuals with ISH and significant structural arterial stiffness, a high PP frequently occurred with a low DBP [3, 50]. MAP often drops under those arterial conditions and results in a lower TPR (see ISH subtype in Table 1). Previous clinical studies in arterial HT have also dealt with this hemodynamic triad of high PP, low MAP and a TPR lower than expected, most likely due to large artery structural stiffness [3, 50]. Likewise, a nondivergent BP profile (with PP < 50 mmHg) is indicative of an adequate C_t_ even in old subjects (Supplementary Fig. 1).

It follows directly from the above discussion that despite the constraints of a 2-WK model, the analysis of SBP and DBP measurements combined with MAP and PP values delivers great insights concerning arterial hemodynamics in a 24 h ABPM. Additionally, regardless of the patient´s age, a 24 h-ABPM profile reflects the state of C_t_ and TPR in arterial HT.

### Limitations

The current C_t_ method does not employ an arterial pressure waveform and, therefore, cannot be used to assess pulse wave velocity and other arterial properties based on arterial waveforms [54]. In particular, central BP and central PP evaluation with our model are not reliable. Other researchers have demonstrated that a three-element Windkessel model outperforms the 2-WK model for central BP assessment [55]. In addition, an arterial model used to estimate central BP usually requires flow waves [56], which we did not use in our study. Nonetheless, the 2-WK model accurately predicts SBP and DBP [26], the key BP values employed in our model for C_t_ assessment.

We could not exclude other forms of secondary HT that may be mixed within the HT subtypes. In particular, renal and other forms of volumetric HT may influence our CO estimates. Additionally, CO dependence on HR could affect its calculation in subjects with extreme HR values (below 60 and over 100 bpm). Although uncommon, those extreme HR instances during a 24 h ABPM may introduce a limitation in our hemodynamic estimates; in such cases, Frank-Starling effects on SV are noticeable and influence CO values. In the same fashion, obesity leads to an increase in CO estimates. Since TPR has an inverse variation with CO values, these effects must be considered during hemodynamic analysis.

Current analysis concentrated in four HT subtypes and did not take into consideration other important BP phenotypes such as white coat, masked or nocturnal hypertension. In addition, antihypertensive therapy have vascular effects, which may affect to some extent the results with the present method.

Last, due to the a priori definition of each HT subtype, whether these patterns of hemodynamic behavior correspond to particular stages of the disease or different clinical hypertensive phenotypes could not be answered with our cross-sectional study. This important question framed by previous studies of HT hemodynamics [57] needs to be addressed in a different longitudinal study.

### Perspectives

This work is an effort to close the gap between HT pathophysiology knowledge and its clinical application. For clinicians, it is important to synthesize readily available clinical data, such as BP and HR, into useful physiological information. In Table 2, we provide a step-by-step example of the hemodynamic calculations, which converts this method in a handy tool. Physiological models are simplified representations of reality that could provide clinicians with information for decision-making without the need for additional noninvasive or invasive direct measurements. The model presented in this study shows the potential for implementing hemodynamic assessment using the widely available oscillometric BP measurements from clinical settings. By now, 24 h ABPM has brought the possibility of identifying different clinical phenotypes of arterial HT (white coat, masked, non-dipping, nocturnal, etc.), and our results additionally allow the identification of circulatory abnormalities to improve the pathophysiological description of essential HT which, may lead to a more precise diagnosis and treatment selection. The current results may open the possibility of having arterial hemodynamics available to clinicians from 24 h ABPM data to add pathophysiological information to the follow-up of HT patients.

## Conclusions

HT subtypes showed distinctive hemodynamics with regard to C_t_ and TPR, which were extracted with BP and HR values readily available from a 24 h ABPM plus anthropometric features. The new method enables the translation of BP data into useful features of arterial function to build a more accurate pathophysiological description of arterial HT with 24 h ABPM.

## Supplementary Information

Supplementary information

## Data Availability

The data that support the findings of this study are available from the corresponding author upon reasonable request.

## References

[1] O’Brien E, Parati G, Stergiou G, Asmar R, Beilin L, Bilo G (2013). European Society of Hypertension position paper on ambulatory blood pressure monitoring. J Hypertens.

[2] Williams B, Mancia G, Spiering W, Agabiti Rosei E, Azizi M, Burnier M (2018). 2018 ESC/ESH Guidelines for the management of arterial hypertension: The Task Force for the management of arterial hypertension of the European Society of Cardiology and the European Society of Hypertension: The Task Force for the management of arterial hypertension of the European Society of Cardiology and the European Society of Hypertension. J Hypertens..

[3] Galarza CR, Alfie J, Waisman GD, Mayorga LM, Camera LA, del Rio M (1997). Diastolic pressure underestimates age-related hemodynamic impairment. Hypertension..

[4] Li W, Ahn AC (2012). Pulsatile hemodynamics of hypertension: systematic review of aortic input impedance. J Hypertens.

[5] Smith BE, Madigan VM (2018). Understanding the Haemodynamics of Hypertension. Curr Hypertens Rep..

[6] Mahajan S, Gu J, Lu Y, Khera R, Spatz ES, Zhang M (2020). Hemodynamic Phenotypes of Hypertension Based on Cardiac Output and Systemic Vascular Resistance. Am J Med.

[7] Weber T, O’Rourke MF, Ammer M, Kvas E, Punzengruber C, Eber B (2008). Arterial stiffness and arterial wave reflections are associated with systolic and diastolic function in patients with normal ejection fraction. Am J Hypertens.

[8] Saugel B, Cecconi M, Wagner JY, Reuter DA (2015). Noninvasive continuous cardiac output monitoring in perioperative and intensive care medicine. Br J Anaesth.

[9] Farasat SM, Morrell CH, Scuteri A, Ting CT, Yin FC, Spurgeon HA (2008). Pulse pressure is inversely related to aortic root diameter implications for the pathogenesis of systolic hypertension. Hypertension..

[10] Mitchell GF (2018). Aortic stiffness, pressure and flow pulsatility, and target organ damage. J Appl Physiol (1985).

[11] Alfie J, Galarza C, Waisman G (2005). Noninvasive hemodynamic assessment of the effect of mean arterial pressure on the amplitude of pulse pressure. Am J Hypertens.

[12] McEniery CM, Wilkinson IB, Avolio AP (2007). Age, hypertension and arterial function. Clin Exp Pharm Physiol.

[13] Romero CA, Alfie J, Galarza C, Waisman G, Peixoto AJ, Tabares AH (2013). Hemodynamic circulatory patterns in young patients with predominantly diastolic hypertension. J Am Soc Hypertens.

[14] Alvarez-Montoya D, Madrid-Munoz C, Escobar-Robledo L, Gallo-Villegas J, Aristizabal-Ocampo D (2021). A novel method for the noninvasive estimation of cardiac output with brachial oscillometric blood pressure measurements through an assessment of arterial compliance. Blood Press Monit.

[15] Bramlage P, Deutsch C, Krüger R, Wolf A, Müller P, Zwingers T (2014). Validation of the custo screen 400 ambulatory blood pressure-monitoring device according to the European Society of Hypertension International Protocol revision 2010. Vasc Health Risk Manag.

[16] Omboni S, Aristizabal D, De la Sierra A, Dolan E, Head G, Kahan T (2016). Hypertension types defined by clinic and ambulatory blood pressure in 14 143 patients referred to hypertension clinics worldwide. Data from the ARTEMIS study. J Hypertens.

[17] Selvaraj S, Steg PG, Elbez Y, Sorbets E, Feldman LJ, Eagle KA (2016). Pulse Pressure and Risk for Cardiovascular Events in Patients With Atherothrombosis: From the REACH Registry. J Am Coll Cardiol.

[18] Asmar R, Vol S, Brisac AM, Tichet J, Topouchian J (2001). Reference values for clinic pulse pressure in a nonselected population. Am J Hypertens.

[19] Bourgeois MJ, Gilbert BK, Von Bernuth G, Wood EH (1976). Continuous determination of beat to beat stroke volume from aortic pressure pulses in the dog. Circ Res.

[20] Liu Z, Brin KP, Yin FC (1986). Estimation of total arterial compliance: an improved method and evaluation of current methods. Am J Physiol.

[21] Du Bois D, Du Bois EF (1989). A formula to estimate the approximate surface area if height and weight be known. 1916. Nutrition.

[22] Chirinos JA, Rietzschel ER, De Buyzere ML, De Bacquer D, Gillebert TC, Gupta AK (2009). Arterial load and ventricular-arterial coupling: physiologic relations with body size and effect of obesity. Hypertension..

[23] World Medical Association Declaration of Helsinki: ethical principles for medical research involving human subjects. Jama. 2013;310:2191-4. 10.1001/jama.2013.281053 24141714

[24] Frank O (1990). The basic shape of the arterial pulse. First treatise: mathematical analysis. 1899. J Mol Cell Cardiol.

[25] Papaioannou TG, Vardoulis O, Stergiopulos N (2012). The “systolic volume balance” method for the noninvasive estimation of cardiac output based on pressure wave analysis. Am J Physiol Heart Circ Physiol.

[26] Stergiopulos N, Westerhof N (1998). Determinants of pulse pressure. Hypertension..

[27] Westerhof N, Lankhaar JW, Westerhof BE (2009). The arterial Windkessel. Med Biol Eng Comput.

[28] Liu SH, Lin TH, Cheng DC, Wang JJ (2015). Assessment of Stroke Volume From Brachial Blood Pressure Using Arterial Characteristics. IEEE Trans Biomed Eng.

[29] Berger DS, Li JK (1990). Concurrent compliance reduction and increased peripheral resistance in the manifestation of isolated systolic hypertension. Am J Cardiol.

[30] Haluska BA, Jeffriess L, Fathi RB, Mottram PM, Carlier SG, Marwick TH (2005). Pulse pressure vs. total arterial compliance as a marker of arterial health. Eur J Clin Invest.

[31] Krzesinski P, Stanczyk A, Gielerak G, Piotrowicz K (2016). The hemodynamic patterns in hypertensive men and women of different age. J Hum Hypertens.

[32] Papaioannou TG, Vavuranakis M, Tousoulis D (2018). Total arterial compliance: An underestimated biomarker. Eur J Prev Cardiol.

[33] Dart AM, Kingwell BA (2001). Pulse pressure-a review of mechanisms and clinical relevance. J Am Coll Cardiol.

[34] Franklin SS, Lopez VA, Wong ND, Mitchell GF, Larson MG, Vasan RS (2009). Single versus combined blood pressure components and risk for cardiovascular disease: the Framingham Heart Study. Circulation..

[35] AlGhatrif M, Strait JB, Morrell CH, Canepa M, Wright J, Elango P (2013). Longitudinal trajectories of arterial stiffness and the role of blood pressure: the Baltimore Longitudinal Study of Aging. Hypertension..

[36] Ma Y, Choi J, Hourlier-Fargette A, Xue Y, Chung HU, Lee JY (2018). Relation between blood pressure and pulse wave velocity for human arteries. Proc Natl Acad Sci.

[37] Pewowaruk RJ, Korcarz C, Tedla Y, Burke G, Greenland P, Wu C (2022). Carotid Artery Stiffness Mechanisms Associated With Cardiovascular Disease Events and Incident Hypertension: the Multi-Ethnic Study of Atherosclerosis (MESA). Hypertension..

[38] Strandberg TE, Salomaa VV, Vanhanen HT, Pitkala K, Miettinen TA (2002). Isolated diastolic hypertension, pulse pressure, and mean arterial pressure as predictors of mortality during a follow-up of up to 32 years. J Hypertens.

[39] Schillaci G, Pirro M, Mannarino E (2009). Assessing cardiovascular risk: should we discard diastolic blood pressure?. Circulation..

[40] Bourdillon MT, Song RJ, Musa Yola I, Xanthakis V, Vasan RS (2022). Prevalence, Predictors, Progression, and Prognosis of Hypertension Subtypes in the Framingham Heart Study. J Am Heart Assoc.

[41] McEvoy JW, Yang WY, Thijs L, Zhang ZY, Melgarejo JD, Boggia J (2021). Isolated Diastolic Hypertension in the IDACO Study: An Age-Stratified Analysis Using 24-Hour Ambulatory Blood Pressure Measurements. Hypertension..

[42] Stergiopulos N, Meister JJ, Westerhof N (1995). Evaluation of methods for estimation of total arterial compliance. Am J Physiol.

[43] Simon AC, Safar ME, Levenson JA, London GM, Levy BI, Chau NP (1979). An evaluation of large arteries compliance in man. Am J Physiol.

[44] Vardoulis O, Papaioannou TG, Stergiopulos N (2012). On the estimation of total arterial compliance from aortic pulse wave velocity. Ann Biomed Eng.

[45] Vennin S, Li Y, Willemet M, Fok H, Gu H, Charlton P (2017). Identifying Hemodynamic Determinants of Pulse Pressure: A Combined Numerical and Physiological Approach. Hypertension..

[46] Scuteri A, Morrell CH, Orru M, Strait JB, Tarasov KV, Ferreli LA (2014). Longitudinal perspective on the conundrum of central arterial stiffness, blood pressure, and aging. Hypertension.

[47] Lund-Johansen P (1980). Haemodynamics in essential hypertension. Clin Sci.

[48] Tanaka H, Heiss G, McCabe EL, Meyer ML, Shah AM, Mangion JR (2016). Hemodynamic Correlates of Blood Pressure in Older Adults: The Atherosclerosis Risk in Communities (ARIC) Study. J Clin Hypertens.

[49] AlGhatrif M, Lakatta EG (2015). The conundrum of arterial stiffness, elevated blood pressure, and aging. Curr Hypertens Rep..

[50] Franklin SS, Gustin WT, Wong ND, Larson MG, Weber MA, Kannel WB (1997). Hemodynamic patterns of age-related changes in blood pressure. The Framingham Heart Study. Circulation..

[51] Mohiuddin MW, Rihani RJ, Laine GA, Quick CM (2012). Increasing pulse wave velocity in a realistic cardiovascular model does not increase pulse pressure with age. Am J Physiol Heart Circ Physiol.

[52] Mitchell GF, Lacourciere Y, Ouellet JP, Izzo JL, Neutel J, Kerwin LJ (2003). Determinants of elevated pulse pressure in middle-aged and older subjects with uncomplicated systolic hypertension: the role of proximal aortic diameter and the aortic pressure-flow relationship. Circulation.

[53] Zachariah JP, Graham DA, de Ferranti SD, Vasan RS, Newburger JW, Mitchell GF (2014). Temporal trends in pulse pressure and mean arterial pressure during the rise of pediatric obesity in US children. J Am Heart Assoc.

[54] Vennin S, Mayer A, Li Y, Fok H, Clapp B, Alastruey J (2015). Noninvasive calculation of the aortic blood pressure waveform from the flow velocity waveform: a proof of concept. Am J Physiol Heart Circ Physiol.

[55] Mariscal-Harana J, Charlton PH, Vennin S, Aramburu J, Florkow MC, van Engelen A (2021). Estimating central blood pressure from aortic flow: development and assessment of algorithms. Am J Physiol Heart Circ Physiol.

[56] Flores Geronimo J, Corvera Poire E, Chowienczyk P, Alastruey J (2021). Estimating Central Pulse Pressure From Blood Flow by Identifying the Main Physical Determinants of Pulse Pressure Amplification. Front Physiol.

[57] Finkielman S, Worcel M, Agrest A (1965). Hemodynamic Patterns in Essential Hypertension. Circulation..

