# Supplementary material for: Hemodynamic profiles of arterial hypertension with ambulatory blood pressure monitoring
Source: Hypertens Res. 2023 Mar 8;46(6):1482–92. doi: 10.1038/s41440-023-01196-z (PMC10239728; doi:10.1038/s41440-023-01196-z)
Supplement: Supplementary file 1 — Supplementary information [file 41440_2023_1196_MOESM1_ESM.docx]

**SUPPLEMENTARY INFORMATION**

**Title:** Hemodynamic profiles of arterial hypertension with ambulatory blood pressure monitoring

**Authors:** Dagnovar Aristizábal-Ocampo^1,2^; Diego Álvarez-Montoya^1^; Camilo Madrid-Muñoz^1^; Simon Fallon-Giraldo^1^; Jaime Gallo-Villegas^1,3^

^1^Centro Clínico y de Investigación SICOR

^2^Cellular & Molecular Biology Unit, Corporación para Investigaciones Biológicas

^3^Facultad de Medicina, Universidad de Antioquia, Medellín (Colombia).

**Corresponding author:** Dagnovar Aristizábal-Ocampo, MD, Cardiologist, Centro Clínico y de Investigación SICOR, Medellín (Colombia); calle 19, Nº 42-40; telephone: 0057-4-6040007; ORCID iD: 0000-0003-4714-2543; E-mail: [dagnovar@une.net.co](mailto:dagnovar@une.net.co)

**SUPPLEMENTARY MATERIAL CONTENT**

**This supplementary material includes:**

**Supplementary methods 1:** Derivation of total arterial compliance formula with the two-element Windkessel model, mean systolic pressure and systolic time.

**Supplementary methods 2:** Mathematical derivation of the theoretical pulse pressure.

**Supplementary methods 3:** Derivation of equations for systolic time, total arterial compliance and cardiac output with Doppler echocardiography.

**Supplementary figure 1:** Two clinical vignettes with counterexamples showing the effects of total arterial compliance in 24-h blood pressure monitoring profile.

**Supplementary methods 1: Derivation of total arterial compliance formula with the two-element Windkessel model, mean systolic pressure and systolic time**

In each heartbeat, the left ventricular stroke volume (SV) flows into the aorta; one part is “stored” (Q_s_), and the rest moves forward (𝑄_𝑓_) to the peripheral circulation; therefore, the total aortic flow (Q) is:

$$Q = Q_{f}+ Q_{s} Equation 1$$

In the two-element Windkessel model (2-WK), Q is calculated as:

$$Q = \frac{MAP}{R}+ C_{t}\times\frac{\Delta P}{\Delta t} Equation 2$$

Where, Q, flow during the ejection period; MAP, mean arterial pressure (for the complete cardiac cycle [T]); R, systemic vascular resistance; C_t_, total arterial compliance; ΔP, pressure change and Δt, change in a small unit of time.

If only the ejection period is considered, ΔP is equivalent to pulse pressure (PP) and Δt to systolic time (T_s_). Additionally, in systole, mean systolic mean (MSP) is used for the calculation instead of MAP; then, Equation 2 can be rewritten as:

$$Q_{sys}= \frac{MSP}{R}+ C_{t} \times\frac{PP}{T_{s}} Equation 3$$

where Q_sys_, systolic flow; R, systemic vascular resistance; PP, pulse pressure; C_t_, total arterial compliance; T_s_, systolic time (the duration of the ejection period) and MSP, mean systolic pressure (the mean pressure during the ejection period obtained with a trapezoidal approach as the average between SBP and MAP). See Alvarez et al. for calculation details (1).

Since SV is equal to Q_sys_ by T_s,_ multiplying each term in Equation 3 by T_s_ results in:

$$SV= \frac{MSP}{R} \times T_{s}+ C_{t} \times PP Equation 4$$

where SV, stroke volume; MSP, mean systolic pressure (the mean pressure during the ejection period); R, systemic vascular resistance; T_s_, systolic time; C_t_, total arterial compliance and PP, pulse pressure. The first term of the sum is the forward volume, which is propelled into the peripheral circulation, and the second term is the transverse or “stored” volume in the elastic aorta. Cardiac output (CO) is obtained as the product of heart rate (HR) and SV.

In the 2-WK model, the diastolic pressure decay time constant (τ) is the product of R and C_t_; therefore, 𝑅 = 𝜏⁄*C****_t_*** (2). Additionally, on physiological grounds, τ is the ratio between the steady and pulsatile blood pressure (BP) components with regard to T (see Supplementary methods 2, which demonstrates mathematical derivation of the theoretical pulse pressure). Thus, 𝑅 = 𝜏⁄*C****_t_*** is substituted for R in Equation 4to derive a formula for C_t_:

$$C_{t} = \frac{SV}{\left( PP+MSP \times\frac{T_{s}}{\tau} \right)} Equation 5$$

where C_t_, total arterial compliance; SV, stroke volume; PP, pulse pressure; MSP, mean systolic pressure (the mean pressure during the ejection period); T_s_, systolic time; and τ, diastolic pressure decay time constant. Equation 5 was used to obtain an estimated value for C_t_ with the reference SV value acquired from Doppler echocardiography (1).

The mathematical expression $\left( PP+MSP \times\frac{T_{s}}{\tau} \right)$ in the denominator of equation 5 corresponds to a theoretical pulse pressure (PPth) in the arterial system without systolic runoff (see Supplementary methods 2, which demonstrates mathematical derivation of the theoretical pulse pressure).

Then,

$$C_{t}= \frac{SV}{PPth} Equation 6$$

where C_t_ is the total arterial compliance; SV is the stroke volume; and PPth is the theoretical pulse pressure. The PPth was the basis for obtaining a reliable C_t_ estimate since the commonly used SV/PP ratio overestimates C_t_ (3), considering that this last ratio ignores the peripheral blood flow during the systolic period (4).

**Supplementary methods 2: Mathematical derivation of the theoretical pulse pressure**

The volume of blood entering the arterial system from the left ventricle (LV) distends the proximal arterial vessels during systole, according to the elastic properties of the aorta. There, one part is “stored” (Q_s_), and the rest moves forward (𝑄_𝑓_) to the peripheral circulation. If the stroke volume (SV) is the sum of the volume of blood draining into the periphery during systole (systolic runoff volume) and the volume stored within the aorta, these two volumes could be defined independently in terms of changes in aortic pressure that occur as a result of their presence (4). Let the change in pulse pressure (ΔPP) represent the additional increment in aortic pressure which would have occurred if peripheral drainage had been prevented during aortic ejection (see **Fig. 1** below). The total pressure equivalent of systolic ejection could be expressed as the sum of these two components, the one “stored” that provokes the pulse pressure (PP) and the systolic runoff that causes the ΔPP. This sum corresponds to theorical pulse pressure (PPth) (4). Physiologically, the PPth is attenuated due to peripheral drainage of blood during any given systole (i.e., the “systolic runoff”) (4).

Consequently, PPth represents the sum of the PP and the systolic runoff pressure.

$PPth=PP+ systolic runoff pressure$ (*Equation 1*)

Initially, Bourgeois estimated the systolic runoff pressure as the ratio of systolic area (SA) of the blood pressure (BP) curve and the time constant (τ) of the decrease of aortic pressure during diastole (4).

$Systolic runoff pressure= \frac{SA}{\tau}$ (*Equation 2*)


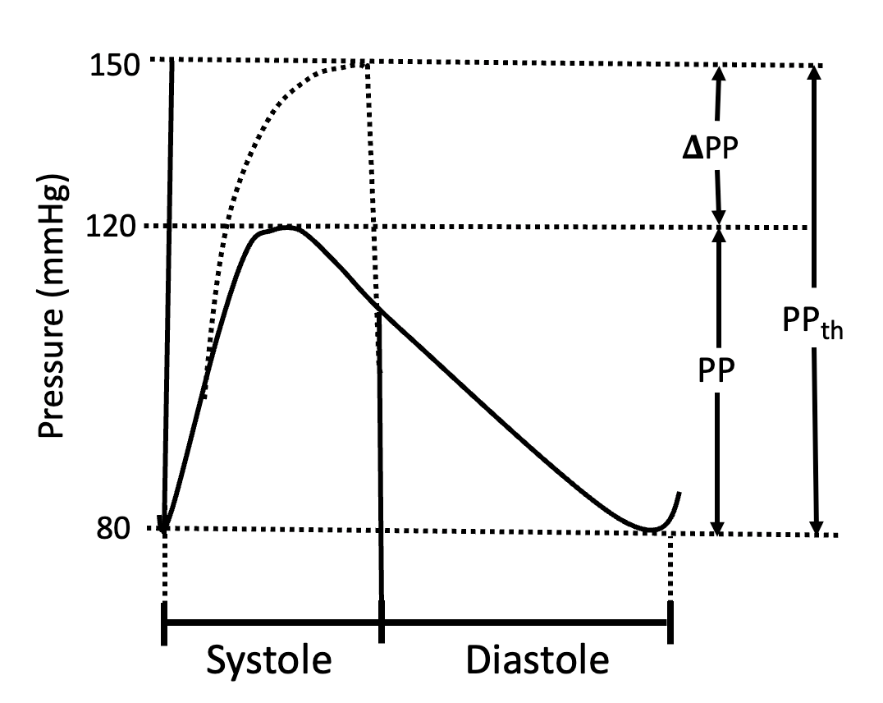


**Fig. 1** Graphical representation to explain the theoretical pulse pressure (PPth). The solid curve is a normal pressure curve from a two-element Windkessel model. The dashed curve above the systolic portion of pressure curve represents the theoretical aortic pressure contour resulting in a pressure increment, ΔPP, which would occur if all peripheral vessels were to be closed during systole, thereby preventing the systolic drainage. Therefore, ΔPP is the systolic runoff pressure. PPth represents the sum of PP and ΔPP. Adapted from Bourgeois MJ, Gilbert BK, Von Bernuth G, Wood EH. Continuous determination of beat to beat stroke volume from aortic pressure pulses in the dog. Circ Res. 1976;39(1):15-24.

In our method (1) we found, SA = MSP x T_s_.

With, MSP, mean systolic pressure and T_s_, systolic time; then, replacing this last expression in Equation 2,

$Systolic runoff pressure=MSP \times\frac{T_{s}}{\tau}$ (*Equation 3*)

In turn, by replacing in Equation 1, the following expression is obtained

$PPth=PP+MSP \times\frac{T_{s}}{\tau}$ (*Equation 4*)

This last expression estimates the highest systolic blood pressure increment in the aorta without systolic runoff. As is can be seen, the PPth corresponds to the denominator in the Equation 5 in Supplementary methods 1; thus:

$C_{t}= \frac{SV}{PP+MSP \times\frac{T_{s}}{\tau}}= \frac{SV}{PPth}$(*Equation 5*)

As it can be observed, the commonly used ratio SV/PP overestimates the total arterial compliance $(C_{t})$since it does not take into account the systolic runoff (4).

Traditionally, the value of τ is estimated as (5, 6):

$\tau= \frac{T_{d}}{Ln \left( \frac{SBP}{DBP} \right)}$ (*Equation 6*)

With, τ, diastolic pressure decay time constant; T_d_, diastolic time (i.e., cardiac period [T] minus systolic time [T_s_]); SBP, systolic blood pressure and DBP, diastolic blood pressure.

This equation is based on the exponential approximation to the pressure decay. 𝜏 is the product of systemic vascular resistance (R) and Ct, therefore, 𝜏 = R x C_t_. Both variables are replaced as follows:

$$\tau=\frac{MAP}{CO}\times\frac{SV}{PP+MSP\times\frac{T_{s}}{\tau}} (Equation 7)$$

With, MAP, mean arterial pressure; CO, cardiac output; R, systemic vascular resistance; SV, stroke volume; PP, pulse pressure.

Replacing CO in Equation 7 by SV/T, then:

$$\tau=\frac{MAP}{\frac{SV}{T}}\times\frac{SV}{PP+MSP\times\frac{T_{s}}{\tau}} (Equation 8)$$

Simplifying and doing algebraic transformations, we obtain,

$$\tau=\frac{MAP\times T-MSP\times T_{s}}{PP} (Equation 9)$$

This is a simpler way to calculate $\tau$. Yet, another view of $\tau$ can be obtained by substituting Equation 5 into Equation 8

$$\tau=\frac{MAP}{\frac{SV}{T}}\times\frac{SV}{PPth} (Equation 10)$$

Simplifying, then

$$\tau=\frac{MAP}{PPth}\times T (Equation 11)$$

Equation 11 is a meaningful expression for clinical use, with great insights into the physiological behavior of the arterial system. $\boldsymbol{\tau}$ **is a time and, in turn,** $\boldsymbol{\tau}$ **is a function of the cardiac period and the ratio of steady and the pulsatile components of blood pressure**.

**Supplementary methods 3: Derivation of equations for systolic time, total arterial compliance and cardiac output with Doppler echocardiography**

Doppler echocardiography measurements were obtained in 230 consecutive normotensive and hypertensive subjects. A systolic time (T_s_) equation was obtained after applying a linear regression model; the following coefficients were derived from whole dataset:

| T_s_ (systolic time) (sec)  N=230 - R^2^=0.53 | **Coeff** | **se** | **P** |
| --- | --- | --- | --- |
| Independent term | 0.20614427 | 0.00848816 | <0.0001 |
| Age (years) | 0.000445593 | 8.1817E-05 | <0.0001 |
| Gender (Male=1, Female=0) | -0.013814467 | 0.00252528 | <0.0001 |
| T: Cardiac period (sec) | 0.1223727 | 0.0084142 | <0.0001 |

Equation could be presented as:

$$T_{s}=0.2061+\frac{\mathrm{Age}}{2244}-\frac{Gender}{72.39}+\frac{T}{8.17} (Equation 12)$$

| Bland-Altman |  |  |  |  |
| --- | --- | --- | --- | --- |
|  |  |  |  |  |
|  | *value* | *s.e.* | *lower* | *upper* |
| mean diff | -1.1368E-16 | 0.00122447 | -0.00241266 | 0.00241266 |
| lower limit | -0.03639646 | 0.00209563 | -0.04052565 | -0.03226727 |
| upper limit | 0.03639646 | 0.00209563 | 0.03226727 | 0.04052565 |
|  |  |  |  |  |
| stdev diff | 0.01856996 |  |  |  |
| sample size | 230 |  |  |  |
| alpha | 0.05 |  |  |  |

Error (mean/sigma) = 5.6%

The total arterial compliance indexed by body surface area (C_t_/BSA) was estimated based on PPth and cardiac periods as follows:

| C_t_/BSA (ml/mHg/m^2^)  N=230 - R^2^=0.79 | **Coeff** | **se** | **P** |
| --- | --- | --- | --- |
| Independent term | -0.43327629 | 0.07617812 | <0.0001 |
| 1/PPth | 38.0769764 | 1.72154971 | <0.0001 |
| T_d_/T | 0.78889085 | 0.13508896 | <0.0001 |

Where BSA is body surface area; PPth corresponds to the theorical aortic pulse pressure, T_d_ is the diastolic period (sec) and T is the cardiac period (sec).

Equation could be presented as:

$$\frac{C_{t}}{BSA}=\frac{38}{PPth}+\frac{4}{5}*\frac{T_{d}}{T}-\frac{3}{7}(Equation 13)$$

Bland-Altman

|  | *value* | *s.e.* | *lower* | *upper* |
| --- | --- | --- | --- | --- |
| mean diff | -1.3371E-16 | 0.00506525 | -0.00998045 | 0.00998045 |
| lower limit | -0.15056107 | 0.008669 | -0.16764227 | -0.13347986 |
| upper limit | 0.15056107 | 0.008669 | 0.13347986 | 0.16764227 |
|  |  |  |  |  |
| stdev diff | 0.07681828 |  |  |  |
| sample size | 230 |  |  |  |
| alpha | 0.05 |  |  |  |

Error (mean/sigma) = 13.9%

The SV was estimated using Equation 6 (Supplementary methods 1) and multiplied by heart rate to obtain the CO. Values obtained were utilized to analyze the agreement between the model and Doppler echocardiography data.

| Bland-Altman CO |  |  |  |  |
| --- | --- | --- | --- | --- |
|  |  |  |  |  |
|  | *value* | *s.e.* | *lower* | *upper* |
| mean diff | 6.39249192 | 47.803222 | -87.7978907 | 100.582875 |
| lower limit | -1414.52604 | 81.8136031 | -1575.72971 | -1253.32237 |
| upper limit | 1427.31102 | 81.8136031 | 1266.10736 | 1588.51469 |
|  |  |  |  |  |
| stdev diff | 724.971756 |  |  |  |
| sample size | 230 |  |  |  |
| alpha | 0.05 |  |  |  |

Error (mean/sigma) = 13.7%

**Supplementary figure 1: Two clinical vignettes with counterexamples showing the effects of total arterial compliance in 24-h blood pressure monitoring profile**

| **Case 1**. A 71 y/o asymptomatic male attends an annual health check-up. Baseline BP was normal; however, he had a hypertensive response during the treadmill stress test. A 24-h ABPM was indicated to clarify possible masked HT. | | **Case 2**. A 58 y/o male with family history of early cardiovascular disease visits his primary care physician for a routine evaluation during which he appears with *de novo* hypertension. A 24-h ABPM is ordered for diagnosis confirmation. | |
| --- | --- | --- | --- |
| **24-h ABPM** | | **24-h ABPM** | |
| 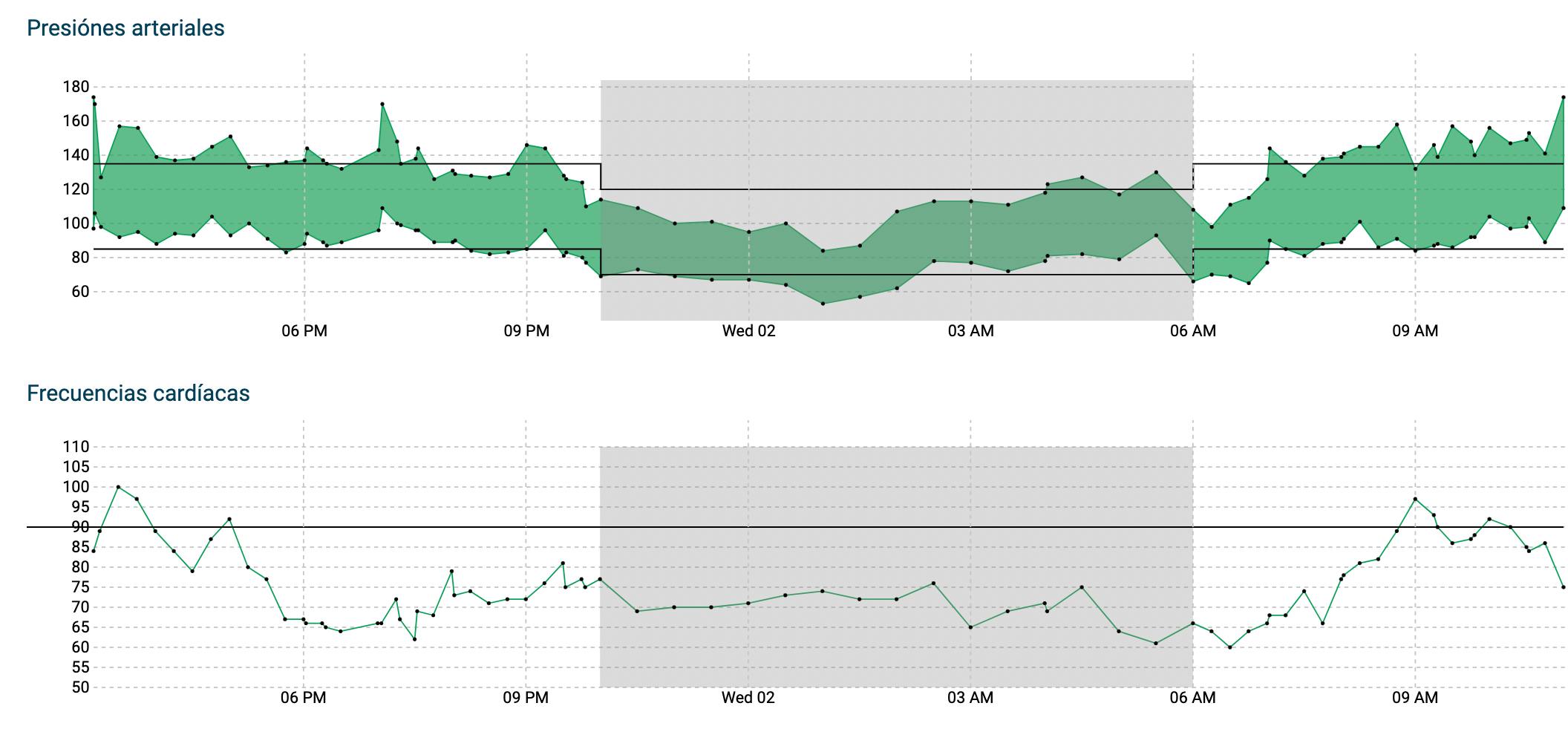 | | 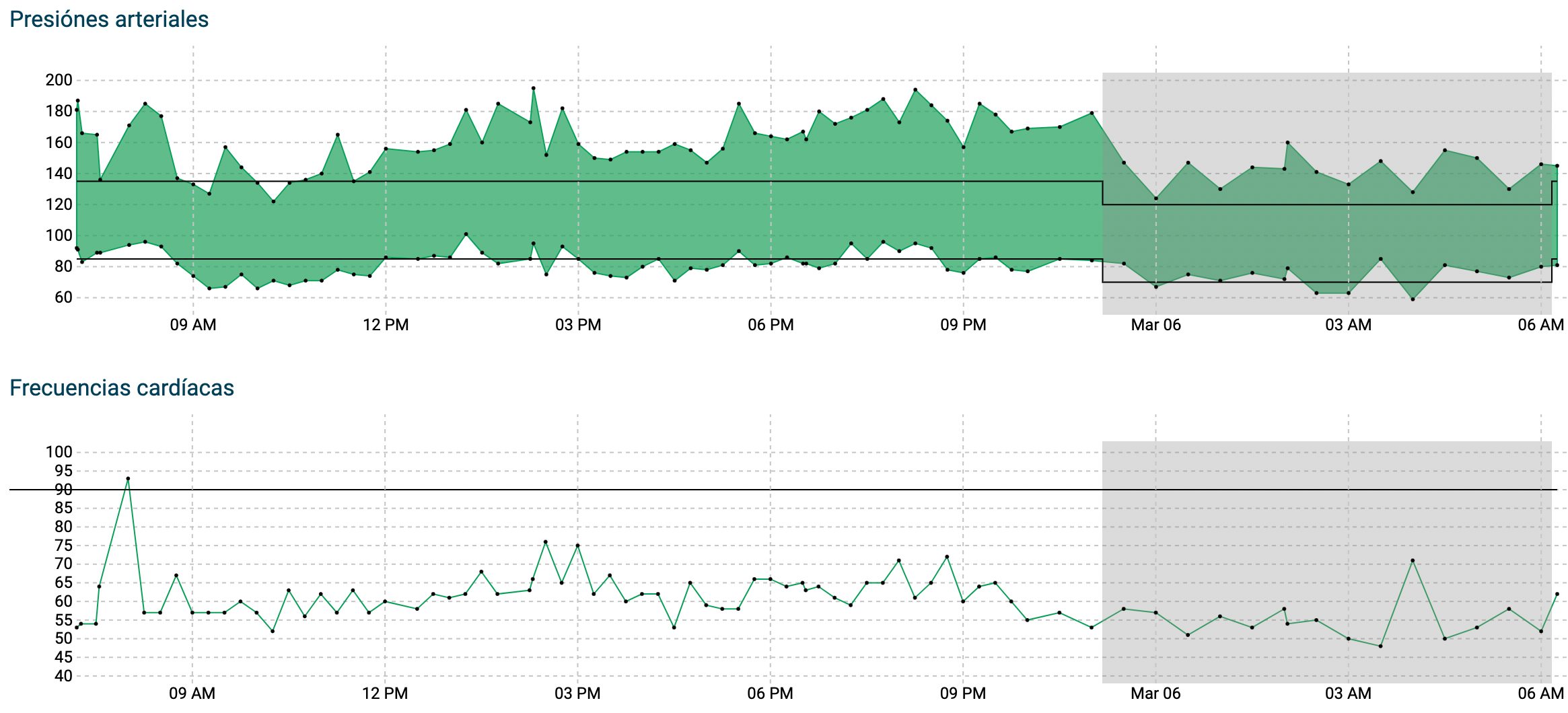 | |
| Average BP (mmHg) | 132/86 | Average BP (mmHg) | 158/80 |
| Average HR (bpm) | 76 | Average HR (bpm) | 61 |
| Mean pressure (mmHg) | 102 | Mean pressure (mmHg) | 107 |
| Pulse pressure (mmHg) | 46 | Pulse pressure (mmHg) | 78 |
| Total arterial compliance (mL/mmHg) | 0.90 | Total arterial compliance (mL/mmHg) | 0.75 |
| **Carotid-femoral pulse wave velocity** | | **Carotid-femoral pulse wave velocity** | |
| 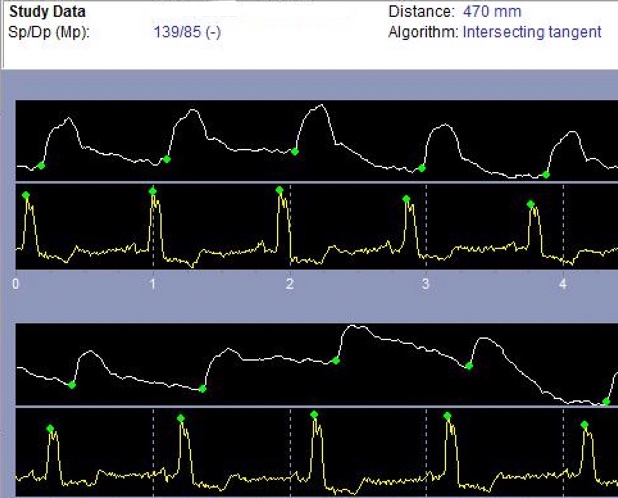 | | 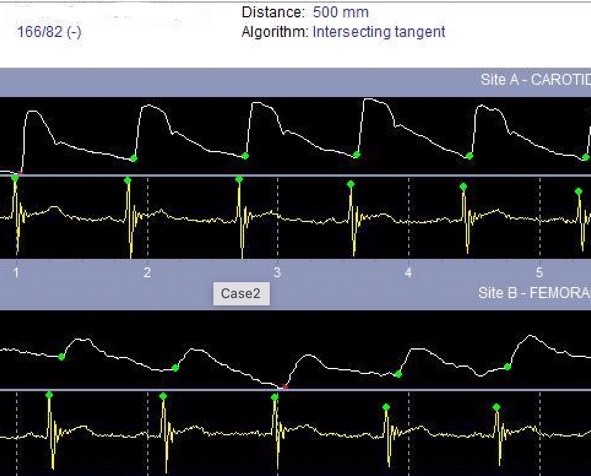 | |
| **Analysis**: A non-divergent systolic-diastolic HT subtype is observed despite patient´s advanced chronological age. Total arterial compliance is 0,90 mL/mmHg with a pulse pressure of 46 mmHg. Carotid-femoral pulse wave velocity of 9,1 m/s indicates no meaningful arterial stiffness. | | **Analysis**: A much younger patient with a divergent blood pressure profile and predominantly systolic hypertension. Pulse pressure is 78 mmHg. Total arterial compliance is 0,75 mL/mmHg (reduced). Carotid-femoral pulse wave velocity of 12,4 m/s revealed a significant arterial stiffness. | |

The total arterial compliance effects appear to be independent of age-related effects on arterial system. BP: blood pressure; 24-h ABPM: 24-h ambulatory blood pressure monitoring; HT: hypertension; HR: heart rate.

**References**

1. Alvarez-Montoya D, Madrid-Munoz C, Escobar-Robledo L, Gallo-Villegas J, Aristizabal-Ocampo D. A novel method for the noninvasive estimation of cardiac output with brachial oscillometric blood pressure measurements through an assessment of arterial compliance. Blood Press Monit. 2021;26(6):426-34.

2. Westerhof N, Lankhaar JW, Westerhof BE. The arterial Windkessel. Med Biol Eng Comput. 2009;47(2):131-41.

3. Stergiopulos N, Meister JJ, Westerhof N. Evaluation of methods for estimation of total arterial compliance. Am J Physiol. 1995;268(4 Pt 2):H1540-8.

4. Bourgeois MJ, Gilbert BK, Von Bernuth G, Wood EH. Continuous determination of beat to beat stroke volume from aortic pressure pulses in the dog. Circ Res. 1976;39(1):15-24.

5. Berger DS, Li JK. Concurrent compliance reduction and increased peripheral resistance in the manifestation of isolated systolic hypertension. Am J Cardiol. 1990;65(1):67-71.

6. Liu Z, Brin KP, Yin FC. Estimation of total arterial compliance: an improved method and evaluation of current methods. Am J Physiol. 1986;251(3 Pt 2):H588-600.
